# Analysis of the Ability to Produce Pleasant Aromas on Sour Whey and Buttermilk By-Products by Mold *Galactomyces geotrichum*: Identification of Key Odorants

**DOI:** 10.3390/molecules26206239

**Published:** 2021-10-15

**Authors:** Kamila Szudera-Kończal, Kamila Myszka, Piotr Kubiak, Małgorzata Anna Majcher

**Affiliations:** Faculty of Food Science and Nutrition, Poznań University of Life Sciences, 60-624 Poznań, Poland; kamila.szudera-konczal@up.poznan.pl (K.S.-K.); Kamila.myszka@up.poznan.pl (K.M.); piotr.kubiak@up.poznan.pl (P.K.)

**Keywords:** whey, buttermilk, *Galactomyces geotrichum*, fermentation, aroma-active compounds, aroma biotechnology, SIDA, GC-O, OAV

## Abstract

Currently, there is a growing demand for flavorings, especially of natural origin. It is worth paying attention to the biotechnological processes of flavor production, characterized by simplicity, high efficiency and relatively low cost. In this study, we analyzed the ability of the *Galac tomyces geotrichum* mold to transform by-products of the dairy industry: sour whey and buttermilk to complex flavour mixtures with pleasant, honey-rose aroma. Furthermore, the aroma complexity of the fermentation product has been carefully identified applying a sensomic approach involving the use of gas chromatography-olfactometry (GC-O), gas chromatography-mass spectrometry (GC-MS) and stable isotope dilution assay (SIDA) to identify and quantify aroma compounds. Based on the calculation of odor activity value (OAV), 13 key aroma compounds were present in both tested variants. The highest OAVs were found for phenylacetaldehyde (honey-like) in the buttermilk variant (912) and 2-phenylethanol (rose-like) in the sour whey variant (524). High values of this indicator were also recorded for phenylacetaldehyde (319) and 3-methyl-1-butanol with a fruity aroma (149) in the sour whey culture. The other compounds identified are 3-methylbutanal (malty), 2,3-butanedione (cheesy), isovaleric acid (cheesy), 3-(methylthio)-propanal (boiled potato), butanoic acid (vinegar), (*E*)-2-nonenal (fatty), ethyl furaneol (burnt sugar), dimethyl trisulfide (cabbage), and acetic acid (vinegar).

## 1. Introduction

Modern food production technologies shape the growing demand for flavorings. This is related to the shortening and simplification of processing, which may result in obtaining an aroma of the product with insufficient intensity. The aroma compounds can be obtained by chemical synthesis, extraction from natural sources, and biotechnological processes. Synthetic aroma compounds are characterized by high production efficiency, simplicity of the technology, and low price. However, they are less and less acceptable among consumers who prefer food additives of natural origin. Moreover, obtaining volatile compounds by means of chemical reactions is very often energy-consuming and harmful to the environment. The extraction of aromas from natural sources, on the other hand, is subject to several limitations, such as the impact of seasonality, climate changes, and political features on the availability of the raw material; the difficulty and time-consuming technologies, or a higher price. In the face of the growing demand for natural aromas, biotechnological processes are a promising alternative to traditional methods. These technologies include de novo synthesis and bioconversion of natural precursors using microbial cells or enzymes. Biotechnological production of flavors presents several important benefits when compared with the previously mentioned methods, such as high production efficiency, simplicity of the technology, controllability, independence from many external factors, care for natural resources, relatively low production cost, and the possibility of managing industry by-products. However, the factors limiting these technologies are the possible variability of the composition of media containing ingredients of natural origin, the need to develop technologies for the recovery of aroma compounds in some cases, as well as the possibility of contamination of the culture by incomplete sterilization of the medium, contamination of the inoculum or careless handling during sampling and other necessary activities [1,2,3,4].

Food is a complex matrix for which several compounds are responsible for the aroma. Therefore, there is a growing interest in producing aroma mixtures rather than individual compounds, as they can more closely resemble the aroma of food resulting from natural transformations [5]. This approach also eliminates the need to implement time-consuming and costly processes to extract specific aroma compounds from microbial cultures. Potential applications of aroma compositions are food products whose aroma is not characterized by a sufficient intensity due to the shortening of fermentation processes, as in the brewing, dairy or baking industries. An important stage in developing new technologies for the biotechnological production of aroma mixtures is the determination of the key odorants in the resulting product due to the multitude of aroma compounds. Previous studies have shown that the best approach to fulfil this task is the application of sensomic concepts, which allows for careful identification of odor-active compounds responsible for the sensory properties and sensory acceptance of fermented products [5,6,7].

There has, in recent years, been a continuous increase in the consumption of milk and dairy products. The growing demand for these products generates an increase in their production, resulting in a greater supply of dairy industry by-products, including whey and buttermilk [8]. Whey is a yellowish liquid obtained during cheese production by separating the curd. Depending on the type of technology, there are two types of whey: sweet whey, formed after enzymatic coagulation, and sour whey, formed during the production of cottage cheese. Whey contains 93–94% of water, 4.5–6.0% of lactose, 0.6–1.1% of proteins, 0.8–1.0% of minerals, 0.05–0.9% of lactic acid, and 0.06–0.5% of fat, depending on the method of obtaining it [9,10]. Buttermilk is a cream-colored liquid, aqueous side stream obtained during butter production by separating the milk fat. This by-product contains 91–92% of water, 3.6–6.7% of lactose, 2.4–3.5% of proteins, 0.6–0.8% of minerals, 0.7% of lactic acid, and 0.5–1.5% of fat, depending on the method of obtaining it [11,12,13]. It should be noted that both whey and buttermilk contain L-phenylalanine, which is a precursor of rose-like aroma compounds formed in the Ehrlich pathway [14,15,16]. In this study, sour whey and buttermilk were analyzed, both in spray-dried form. This approach is due to the wide availability of these by-products in this form and the possibility of easier and longer storage and lower transport costs. The key factor, however, was the ability to sterilize the spray-dried medium components by exposure to UV radiation, which prevents changes in the final aroma due to thermal reactions. According to FAOSTAT [17], the global production of spray-dried sour whey and buttermilk in 2018 amounted to over 2.8 and almost 0.7 million tones, respectively. Over the ten years, an increase in production was observed in the amount of over 0.3 and 0.5 million tons, respectively.

*Galactomyces geotrichum* is a mold that is part of the natural microflora of raw milk and dairy products, affecting the chemical composition as well as nutritional and sensory properties of the resulting food products [18,19]. It should be noted that *G. geotrichum* mold is Generally Recognized as Safe (GRAS), so there is no risk of potential toxicity from this microorganism [20]. According to our recent studies, *G. geotrichum* is responsible for the formation of intense aroma with honey, rose and fruity odor notes on substrates with sweet whey and sour whey [21].

In the present studies, the ability of *G. geotrichum* mold to transform by-products of the dairy industry (sour whey and buttermilk) to complex flavour mixtures was investigated. This study is part of a larger experiment to analyze the aroma produced by *G. geotrichum* on different substrate variants containing dairy by-products [21]. The aroma composition of obtained post-fermentation product was fully characterized using a sensomic approach including gas chromatography olfactometry (GC-O) analysis, solvent assisted flavour evaporation (SAFE) extraction, gas chromatography and mass spectrometry (GC/MS), and calculation of odor activity values (OAV).

## 2. Results and Discussion

### 2.1. Optimization of Culture Conditions

This experiment aimed to select conditions for the cultivation of *G. geotrichum* mold on media with sour whey and buttermilk, which would allow for the production of the pleasant honey-rose aroma of the highest possible intensity. Therefore, four variants of sugar added to the medium, three variants of the pH value, and three variants of the incubation temperature were analyzed. Each of the tested variants was subjected to a profile sensory analysis, including the evaluation of the intensity of four descriptors: honey-like, rose-like, fruit-like, and caramel-like, and general desirability, meaning whether the aroma is liked or not.

#### 2.1.1. Type of Sugar

**Figure 1:**
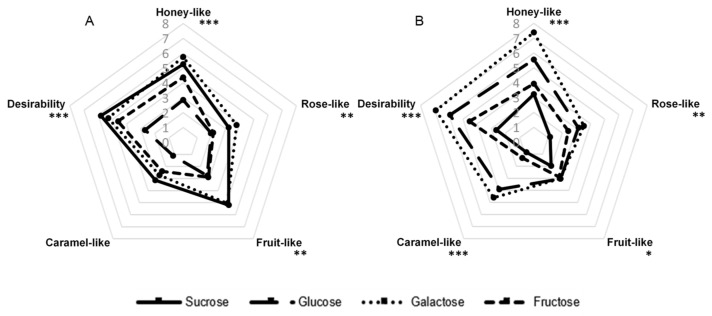
Sensory profiles of post-fermentation products from flask cultures with (**A**) sour whey and (**B**) buttermilk for various types of sugars. * *p* < 0.05; ** *p* < 0.01; *** *p* < 0.001.

The influence of the addition of the following sugars on the sensory quality of the aroma produced by *G. geotrichum* was analyzed: sucrose, glucose, galactose, and fructose. Lactose was not included in this experiment because our preliminary studies showed that *G. geotrichum* mold is incapable of fermenting it. The results in Figure 1 illustrate that the variants with galactose as a component of the medium of both products and the variant with sucrose in the case of sour whey had the most extensive aroma profiles. The post-fermentation product with sour whey and sucrose as ingredients in the culture medium was subjected to a detailed analysis of key odorants in our previous studies [21]. The same variants were characterized by the highest general desirability (5.8). The fermentation product with sour whey and galactose gave strong honey-like (5.7), rose-like (3.8), and fruit-like (5.1) notes. In the case of the buttermilk product with the addition of galactose, the panelists did not find a strong fruit-like aroma but assessed the caramel-like note (4.6) as intense. The glucose variant had a similar odor profile but with a lower intensity. Moreover, the post-fermentation product with buttermilk and galactose was characterized by higher desirability (6.9) and intensity of the honey-like aroma note (7.4) than any sample with sour whey. The sensory profile of both fructose-containing variants was assessed as significantly less developed. The variant with sour whey and glucose and the variant with buttermilk and sucrose were characterized by the least intense aroma.

**Figure 2:**
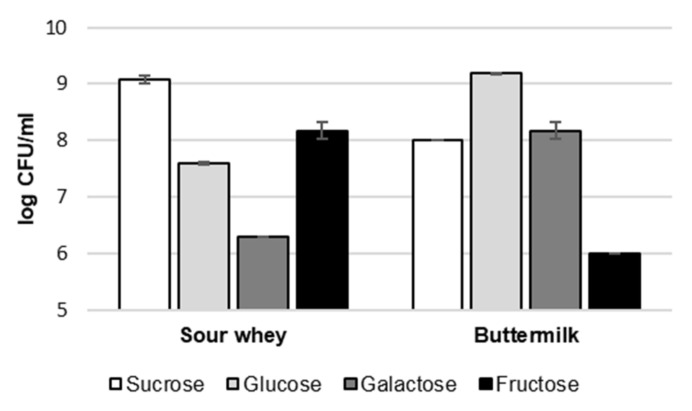
The concentration of biomass in post-fermentation products from flask cultures with sour whey and buttermilk for various types of sugars. Error bars represent ± SD from the replicates.

The number of CFUs was determined in all analyzed samples to investigate the influence of the amount of *G. geotrichum* biomass on the intensity of specific aroma notes. However, the results in Figure 2 show no correlation between the biomass concentration and the intensity of the aroma. In the case of sour whey, as a nutrient component, the samples with the most extensive and at the same very similar odor profile (galactose and sucrose) showed extremely different results in terms of CFU concentration of *G. geotrichum*. The variant with galactose was characterized by the lowest concentration of biomass (6.3 log CFU/mL), while the variant with sucrose—the highest (9.1 log CFU/mL). In the case of buttermilk, the variants with the broadest aroma profiles (galactose and glucose) were characterized by a high biomass concentration (8.2 and 9.2 log CFU/mL, respectively). However, a high number of CFU *G. geotrichum* was also found in the variant with sucrose (8.0 log CFU/mL), in which no high-intensity notes were found.

#### 2.1.2. pH Value

**Figure 3:**
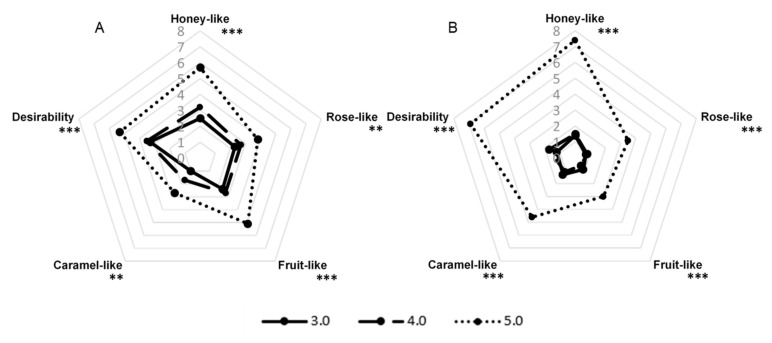
Sensory profiles of post-fermentation products from flask cultures with (**A**) sour whey and (**B**) buttermilk for various types of PH of the media. ** *p* < 0.01; *** *p* < 0.001.

*G. geotrichum* grows over a broad PH range; however, 5.0–5.5 are considered optimal [22]. We tested three variants of the PH of the media, determined at the beginning of the culture, to find the variant with the broadest aroma profile. The results in Figure 3 show that, for both sour whey and buttermilk cultures, the aroma of the highest intensity was observed at the PH value of 5.0. The aroma profile of the post-fermentation product formed on the sour whey medium with PH 4.0 was about half as intense, and the variant with PH 3.0 was characterized by the lowest intensity. In the case of buttermilk, panelists evaluated variants with PH 3.0 and 4.0 as poorly perceptible.

#### 2.1.3. Incubation Temperature

**Figure 4:**
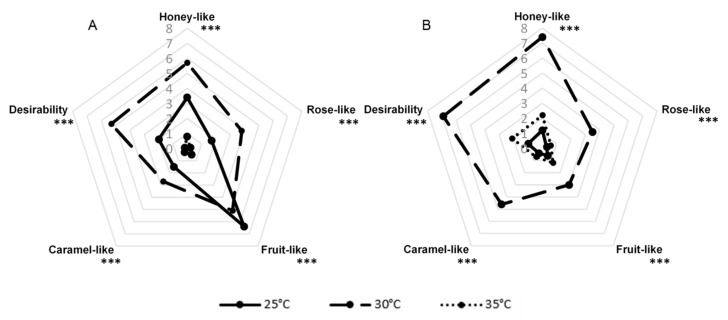
Sensory profiles of post-fermentation products from flask cultures with (**A**) sour whey and (**B**) buttermilk for various incubation temperatures. *** *p* < 0.001.

*G. geotrichum* has been shown to be able to grow over a wide temperature range of 5–38 °C during cheese production, with 25–30 °C being given as the optimal growth temperature [22]. The comparison of three variants of incubation temperatures (25, 30, and 35 °C) is presented in Figure 4. The results showed that the broadest aroma profile was obtained after a 7-day culture at 30 °C for sour whey and buttermilk product. After growing *G. geotrichum* on the sour whey medium at 25 °C, panelists noted an intense note of rotten fruit. The variants incubated at 35 °C in the case of sour whey and 25 °C in the case of buttermilk had a very slightly perceptible aroma.

#### 2.1.4. Determining of Culture Conditions

Based on the results of the sensory evaluation, the following optimal conditions were selected for the culture of *G. geotrichum* mold on substrates with sour whey and buttermilk: galactose as the type of sugar in the medium, medium PH at 5.0, and an incubation temperature of 30 °C.

### 2.2. Sensory Evaluation of Flask Cultures

**Figure 5:**
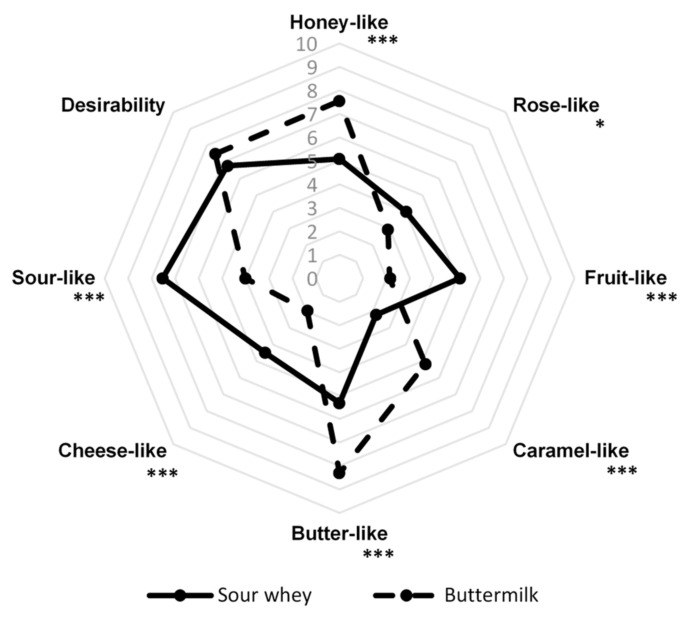
Sensory profiles of post-fermentation products from flack cultures with sour whey and buttermilk. * *p* < 0.05; *** *p* < 0.001.

After determining the optimal conditions for the cultivation of *G. geotrichum* on media with sour whey and buttermilk, flask cultures were carried out. The obtained post-fermentation products were subjected to a sensory evaluation, including additional odor descriptors, the results of which are presented in Figure 5.

The desirability of both products was highly appreciated by the panelists, despite the fact that their sensory profiles are very diverse. The slightly higher acceptability of the post-fermentation product with buttermilk (7.5) than the variant with sour whey (6.8) was probably due to the strong honey aroma desired by consumers. The variant with the addition of sour whey was characterized by a high intensity of honey-like (5.1), rose-like (4.0), fruit-like (5.1), butter-like (5.3), and cheese-like (4.5) notes. The panelists evaluated the intensity of the sour-like note (7.5) as the highest, while the caramel aroma (2.2) was the least perceptible. In the case of products with the addition of buttermilk, a much greater intensity of honey-like (7.6), caramel-like (5.2), and butter-like (8.3) notes were observed than in the case of sour whey variant. There was also a sour-like aroma (4.0) in this product. Rose-like (2.9), fruit-like (2.1), and cheese-like (2.0) notes were the least perceptible. The desirability of both products was highly appreciated by the panelists.

The presence of a honey-rose aroma is found in many products, which includes fermentation processes, such as cheese, pumpernickel bread or traditional pastes [19,23,24,25]. It has been well established that the most frequently responsible for this aroma are compounds formed during the transformation of L-phenylalanine in the course of the Ehrlich pathway, such as phenylacetaldehyde, 2-phenylethanol, phenylacetate, and phenylacetic acid [15,26,27]. According to Dunkel et al. [5], phenylacetaldehyde and 2-phenylethanol have been identified as key odorants in over 50 different food products (51 and 52 respectively), such as alcoholic beverages, fermentation products or thermally treated foods. In previous studies, we have shown that 39 strains of *G. geotrichum* are capable of producing phenylacetaldehyde and 2-phenylethanol on a basic medium (without the addition of whey or buttermilk) [21]. The use of appropriate additives to the medium and the optimization of culture conditions allowed for obtaining the post-fermentation product with an extensive, pleasant honey-rose aroma profile.

### 2.3. Identification of Key Aroma Compounds in a Post-Fermentation Product Using GC−O Analysis and Calculation of OAVs

In order to fully characterize the post-fermentation products obtained in the course of biotechnological transformation, the key aroma compounds were identified using GC-O analysis and OAVs calculation. In SAFE extracts prepared from the samples obtained from the sour whey and buttermilk media after the flask cultures with *G. geotrichum* using the parameters defined during the culture optimization, 13 aroma compounds were identified during the GC-O analysis. Table 1 presents the identified compounds with the results of their quantitation and the OAVs. According to Schieberle [28], OAV can be used as an indicator of the aroma compound activity and is calculated by dividing the concentration of an analyte by its odor threshold (OT) value. Choosing an appropriate matrix for OT is crucial as it influences the release of volatile compounds. As the matrix of the post-fermented product contains mostly water, the OT used for calculation in our studies is also based on water. The calculation of OAVs allows for determining the influence of individual compounds on the overall aroma of a product [6,29,30,31,32]. The higher the value of this indicator, the greater the importance of the analyzed volatile in shaping the aroma. The results of the OAV calculations obtained in our studies indicate that all quantified compounds are characterized by OAVs greater than 1.0, which indicates their potential in the aroma formation of the analyzed products. It should be noted that 8 of the identified compounds (2,3-butanedione, acetic acid, 3-methylbutanal, butanoic acid, isovaleric acid, 3-(methylthio)-propanal, furaneol, and (*E*)-2-nonenal) belong to the group of compounds called “generalist”, which are key odorants in more than 25% of food products [5]. In the sour whey samples, two compounds were found having OAVs ranging from 0 to 10, six compounds ranging from greater than 10 to 100, two ranging from greater than 100 to 500, and one compound ranging from greater than 500 to 1000. In the buttermilk variant, two compounds were found having OAVs ranging from 0 to 10, nine compounds ranging from greater than 10 to 100, and one compound ranging from greater than 500 to 1000. One compound was identified with a concentration below the detection limit in both variants, which made it impossible to calculate OAV.

The results presented in Table 1 show that the compounds with the highest OAVs were 2-phenylethanol with rose aroma (524) and phenylacetaldehyde (319) in the sour whey variant, and phenylacetaldehyde with honey aroma (912) in the buttermilk post-fermentation product. It should be noted that 2-phenylethanol was found in the second highest concentration produced by *G. geotrichum* on the sour whey substrate. At the same time, phenylacetaldehyde did not have such a high concentration in any tested variants. This can be explained by 60 times higher phenylacetaldehyde odor threshold (in water) than 2-phenylethanol [33]. Phenylacetaldehyde can be formed either during heat treatment via *Strecker* reaction between dicarbonyl compounds and amino acids or via the *Ehrlich* pathway through transamination and decarboxylation of amino acid. In our studies, we have used the procedures limiting the possibility of Maillard reactions during the preparation of culture media and the fermentation process by *G. geotrichum* to ensure that the compounds in the analyzed post-fermentation product are formed strictly upon bioconversion. Microbial metabolism involves the formation of phenylacetaldehyde and 2-phenylethanol during the Ehrlich pathway involving transamination of L-phenylalanine to phenylpyruvate, followed by its decarboxylation to phenylacetaldehyde, which is then reduced to 2-phenylethanol [15,27]. Due to the differences in OT value between those two compounds, the higher the content of phenylacetaldehyde, at the expense of 2-phenylethanol, the higher aroma intensity of the final product. This has been exemplified by the post-fermentation product made from buttermilk. Although the concentration of 2-phenylethanol was higher than of phenylacetaldehyde (4653 and 3650 µg/kg, respectively), the OAV values showed that phenylacetaldehyde has the biggest influence on the aroma formation as its OAV was 912 while for 2-phenylethanol was only 19. These results could also explain the strongly perceived honey-like aroma in buttermilk post-fermentation product during sensory evaluation. In the case of the sour whey variant, the highest OAV value was obtained for 2-phenylethanol (524), which is most likely responsible for the rosy-like aroma perceived by the sensory panel. As for phenylacetaldehyde, based on obtained OAV (319), it is the second most intense compound in the sour whey post-fermentation product, responsible for the honey-like aroma.

The previously discussed compounds have the greatest influence on the aroma produced by the *G. geotrichum* molds due to the highest OAV values. However, the overall sensory characteristics of the resulting post-fermentation products also depend on the remaining 11 compounds identified. Another compound with a high OAV is 3-methyl-1-butanol with a fruity aroma. This compound was characterized by the highest concentration (146223 µg/kg) among all compounds identified in the aroma of the variant with sour whey. Based on its OT, 3-methyl-1-butanol reached OAV of 149 and most likely is responsible for the strong, fruity aroma in sour whey post-fermentation product. In the buttermilk based product, 3-methyl-1-butanol was present at a lower concentration of 2730 µg/kg, resulting in an OAV of 2.8 and a much less intensive fruity aroma in the sensory profiles. 3-methyl-1-butanol is produced during the fermentation process in beverages such as wine and cider [34,35,36]. Moreover, in the case of wine, its formation is related to the microflora naturally present on the grapes [35]. 3-methyl-1-butanol is also produced during the fermentation of sourdough, e.g., in a three-stage process during the production of pumpernickel bread [24]. The next aroma active compound, 3-methylbutanal, with a malty odor was found in both fermentation products at similar concentration levels: 38 µg/kg for sour whey and 36 µg/kg for the buttermilk variant. This compound is known to have a relatively low OT value (0.5 µg/kg in water). Therefore, it has been identified as a key odorants in many food products, such soy sauce, beer, and bakery products such as rye bread and pumpernickel bread [5,24,32,37]. Both 3-methyl-1-butanol and 3-methylbutanal are formed during the transformation of leucine [5,32,38]. 2,3-butanedione is the compound responsible for the strong buttery aroma in samples with buttermilk, where it was present at almost twice the concentration (680 µg/kg) than in the post-fermentation product with sour whey (370 µg/kg). This compound is formed during carbohydrate metabolism of a typical microflora of dairy products fermented by bacteria or molds, such as fried cottage cheese, Camembert, Cheddar, Emmental, and Lazur [5,19,25,39,40]. Two compounds with cheesy odor notes were found in the analyzed products: isovaleric acid and butanoic acid. In the variant with sour whey, which showed a stronger cheesy aroma, they were present at higher concentrations (19153 µg/kg and 32,040 µg/kg, respectively) than in the case of buttermilk (6286 and 13,030 µg/kg). This resulted in the corresponding OAV’s; 39 and 32 for sour whey post-fermentation product and 13 and 13 for buttermilk one. These compounds can be formed by microbial metabolism—isovaleric acid is formed during the transformation of amino acids (isoleucine and leucine) and butanoic acid during the transformation of carbohydrates and triglycerides [5,41]. When considering the methods of whey management, Prazeres et al. [10] presented the use of controlled whey fermentation processes to produce butanoic and acetic acid. Acetic acid was also found in both tested variants. This compound was present in the post-fermentation product with sour whey and buttermilk, in high concentrations (127,250 and 189,780 µg/kg, respectively); however, due to the very high value of OT, it had the lowest impact on the overall odor profiles. The seventh compound with the highest OAV in the sour whey culture and the fourth in the buttermilk variant was 3-(methylthio)-propanal with a boiled potato aroma. This volatile is formed during the transformation of methionine, also as part of microbial metabolism [5]. Another key odorant in order of OAVs is (*E*)-2-nonenal with a fatty smell, found in both the sour whey and buttermilk variants at 3.4 and 3.5 µg/kg, respectively. This compound has been already identified in a drink prepared from Darjeeling black tea or wheat bread, and is formed from unsaturated fatty acids by oxidation of fats. Two compounds are responsible for the caramel flavor in the buttermilk variant: ethyl furaneol (4-hydroxy-5-ethyl-2-methyl-3(2H)-furanone) with the aroma of burnt sugar and furaneol (4-hydroxy-2,5-dimethyl-3(2H)-furanone) with the aroma of caramel. Ethyl furaneol was found in this product at a concentration of 326 µg/kg with OAV 19, while the sour whey variant contains 12 times less of this compound. This volatile is one of the 12 most important odorants that shape the flavor of Japanese soy sauce, and biosynthesis by yeast was indicated as its method of production, suggesting the pentose-phosphate cycle involved in this process [32,42]. This compound is also found in meat sauce, which belongs to the same group of foods as soy sauce [43]. Furaneol was identified in the analyzed samples at the GC-O analysis stage, but the concentrations of this compound turned out to be below the limits of detection. Based on the Dunkel et al. review [5], this compound is a key odorant in 40.5% of foods, contributing to the aroma of soy sauce, various types of bread, and cocoa beans, among others [24,30,32,37]. Furaneol is produced by Maillard reactions and is found in many products subjected to thermal processes; however, Hecquet et al. [44] showed that it can also be biosynthesized by microorganisms [45]. The last identified compound is dimethyl trisulfide with a cabbage odor, which was found in the analyzed samples at the lowest concentrations of the 13 key odorants, 0.6 µg/kg for the sour whey variant and 1.5 µg/kg for buttermilk and OAVs od 6 and 15, respectively. However, no such odor notes have been identified in the post-fermentation products obtained.

The aroma profiles of the analyzed post-fermentation products can be combined with the presence of specific compounds. However, it should be noted that the overall aroma of a mixture of many compounds is not just the sum of the intensity of their individual aroma descriptors [5,46]. The sense of aroma is influenced by olfactory coding (still poorly understood) and olfactory perception, the understanding of which has been greatly improved in recent years [47]. It has been shown that, for as little as four compounds that create the aroma mixture, a loss of their individual aroma notes can be observed. In the analyzed post-fermentation products with sour whey and buttermilk, 13 key odorants were identified. Therefore, they can be called a complex mixtures of aroma compounds, similarly to most aromas of natural origin, which is why the issue of their perception is important [5]. For example, phenylacetaldehyde itself has a honey-like aroma, while when found in cheese in the presence of phenylacetic acid, it contributed to the formation of rose-like/floral notes of greater intensity [25]. Therefore, in addition to analyzing the combination of specific aroma notes with specific compounds, it is so important to perceive the aroma mixture as a whole.

## 3. Materials and Methods

### 3.1. Chemicals

Yeast extract, sucrose, glucose, and medium with chloramphenicol were obtained from BTL (Łódź, Poland). Galactose was purchased from Acros Organic (Geel, Belgium). Citric acid, Na_2_HPO_4_·2H_2_O, and MgCl_2_ were obtained from POCH (Gliwice, Poland). Fructose was obtained from Chempur (Piekary Śląskie, Poland). L-Phenylalanine, lactic acid, sodium sulfate, ethyl acetate, dichloromethane, and diethyl ether were purchased from Sigma-Aldrich (Poznań, Poland). Inulin was obtained from Hortimex Plus (Konin, Poland). Spray-dried sour whey and buttermilk were purchased from Laktopol (Suwałki, Poland). The following reference aroma compounds were purchased from Sigma-Aldrich (Poznań, Poland): 2,3-butanedione, acetic acid, 3-methylbutanal, 3-methyl-1-butanol, butanoic acid, isovaleric acid, 3-(methylthio)-propanal, dimethyl trisulfide, phenylacetaldehyde, furaneol, maltol, 2-phenylethanol, ethyl furaneol, (*E*)-2-nonenal, and phenylacetic acid. The following stable isotopes were obtained from AromaLAB (Freising, Germany): [^13^C_4_] 2,3-butanedione, [^13^C_1_] acetic acid, [^2^H_3_] 2-methylbutanal, [^2^H_2_] 3-methyl-1-butanol, [^2^H_7_] butanoic acid, [^2^H_2_] isovaleric acid, [^2^H_5_] 3-(methylthio)-propanal, [^2^H_6_] dimethyl trisulfide, [^2^H_5_] phenylacetaldehyde, [^13^C_2_] furaneol, [^2^H_3_] maltol, [^2^H_5_] 2-phenylethanol, [^2^H_2_] (*E*)-2-nonenal, and [^2^H_8_] naphthalene.

### 3.2. Microorganisms

The strain 32 *G. geotrichum* was isolated from Wielkopolski fried cottage cheese produced in the vicinity of Poznań in Poland during the ripening stage. Identification of the isolated strain was performed by amplification of the 18SrRna coding sequence. The strain was protected by lyophilization to standardize the inoculum and then deposited in the microbial culture collection of the Food Volatilomics and Sensomic Group.

#### Preparation of the *G. geotrichum* Strain

Strain 32 was lyophilized to standardize inoculum concentration. Shake flask cultivations were carried out in four 300 mL Erlenmeyer flasks with 100 mL of base medium containing per liter (modified, based on Grygier et al. [48]) 22.8 g of Na_2_HPO_4_·2H_2_O, 10.3 g of citric acid, 0.5 g of MgCl_2_, and 0.17 g of yeast extract. The medium was sterilized, and then sucrose and L-phenylalanine were added to it at a concentration of 60 g/L and 21 g/L, respectively. These ingredients were previously exposed to UV radiation for 30 min. This approach aimed to prevent changes in the aroma profile of the products due to the influence of high temperature on the components of the medium. The medium used was also employed in all subsequent experiments. Each flask was inoculated with 10 μL of *G. geotrichum* strain 32, which had previously been revived by inoculating the agar slant with chloramphenicol (incubation at 30 °C for 72 h under aerobic conditions). Fermentation in flasks with analyzed *G. geotrichum* strain was carried out in a GFL 1086 water bath (GFL, Lauda-Königshofen, Germany) at 30 °C for 7 days with shaking (150 rpm). After the fermentation process was completed, the cultures were centrifuged (Biofuge Primo R, Heraeus, Hanau, Germany) for 10 min at 2012.4× *g* (3000 rpm) and 20 °C. The *G. geotrichum* precipitate was resuspended in 400 mL of the mixture of base medium and 10% inulin solution (1:1, *v*/*v*). To determine the number of colony-forming units (CFUs), the culture was sequentially diluted and plated onto agar plates with chloramphenicol, which were incubated for 72 h in 30 °C. After incubation of the agar plates, the CFUs were counted, and the number of colonies per milliliter of culture was calculated. The experiment was performed in triplicate. The freeze-drying process was conducted in a Beta 1–16 freeze dryer (Martin Christ, Osterode am Harz, Germany). The process was initiated with a freezing step at −35 °C for 2 h, followed by the main drying stage at 15 °C for 20 h, and the final drying at 22 °C (5 h). *G. geotrichum* lyophilisates were collected in glass jars under a nitrogen atmosphere.

### 3.3. Determining the Optimal Culture Conditions

The culture conditions were optimized by analyzing the influence of the type of sugar, the PH of the medium and the incubation temperature on the sensory profile of the two variants of the aroma produced by *G. geotrichum*. Both variants were prepared on the base medium. The first one was enriched with sour whey and the second one with buttermilk (spray-dried, at a concentration of 130 g/L). Moreover, at this stage, citric acid was replaced as a component of the nutrient medium with lactic acid, which occurs naturally in both acid whey and buttermilk [9,11]. This approach was aimed at mapping the natural environment of the development of *G. geotrichum*—dairy products [18,19]. Lactic acid was used to adjust the PH of the culture to a predetermined value in the final step of the medium preparation. This action was performed under sterile conditions in the presence of litmus papers as PH indicators. Flask cultures were carried out in 300 mL Erlenmeyer flasks with 200 mL of different medium variants, in triplicate. Each flask was inoculated with 3.4 × 10^6^ CFU of lyophilized *G. geotrichum* strain.

#### 3.3.1. Type of Sugar

The influence of four types of sugar (sucrose, glucose, galactose, and fructose) on the aroma profile of the post-fermentation products was analyzed. In order to avoid the influence of high sterilization temperatures on aroma profiles of the resulting products, each type of sugar was exposed to UV radiation for 30 min and then added to the sterile medium at a concentration of 60 g/L. The PH of the medium was adjusted to 5.0 prior to inoculation with *G. geotrichum*. Flask cultivations were carried out in a water bath at 30 °C for 7 days with shaking (150 rpm). After fermentation, the number of colony forming units (CFU) in each analyzed variant was determined. The sample was successively diluted and plated on chloramphenicol agar plates in triplicate. The plates were incubated for 72 h at 30 °C, and then the CFU were counted to calculate the number of *G. geotrichum* colonies per milliliter of sample.

#### 3.3.2. pH Value

The effect of the PH of the medium on the aroma produced by *G. geotrichum* was examined by fermentation on three variants of media: PH 3.0, 4.0, and 5.0. The type of sugar in the medium was the one selected when optimizing the culture conditions in this direction. The fermentation process was carried out in a water bath at 30 °C for 7 days with shaking (150 rpm).

#### 3.3.3. Incubation Temperature

The influence of the incubation temperature on the aroma profile of the post-fermentation products was analyzed by cultivating the *G. geotrichum* mold in three temperature variants: 25, 30, and 35 °C. The type of sugar in the medium was the one selected when optimizing the culture conditions in this direction, and the pH value was 5.0. Fermentation was carried out in a water bath for 7 days with shaking (150 rpm).

### 3.4. Sensory Evaluation

Descriptive sensory evaluation was carried out by ten experienced panelists experienced in conducting this type of research. The odor descriptors assessed were selected from the basic flavor descriptive language (Givaudan Roure Flavor [49]) and were determined in preliminary tests. The sensory analysis included the following odor descriptors: honey-like, caramel-like, rose-like, and fruit-like. The general desirability of the aroma produced by *G. geotrichum* was also analyzed. Sensory analysis was performed by scoring odor descriptors on a 10 cm linear scale, with the beginning labeled “none” and the end labeled “ very strong”. Panelists were presented with 20 g of samples obtained after fermentation carried out in all analyzed variants of culture conditions. The samples were placed in 100 mL glass containers and maintained at room temperature during analysis. The sensory evaluation of samples obtained from flask cultures after fermentation under the conditions obtained in the optimization process was carried out in the same way, except that, in addition to the previously mentioned aroma notes, the following descriptors were also analyzed: sour-like, butter-like, and cheese-like. The results were converted into numerical values for data analysis. The evaluations were performed in triplicate in separate profile sessions. The values given by panelist were averaged and analyzed by *t*-test. Significant differences were discussed in the text.

### 3.5. Flask Cultures

In order to obtain post-fermentation products on sour whey and buttermilk substrates by *G. geotrichum* mold, flask cultures were carried out using culture parameters determined in relation to the results obtained during the optimization of the culture conditions. The remaining aspects concerning the preparation of the culture medium and the course of fermentation were analogous to the optimization of the culture conditions. Shake flask cultivations were performed in triplicate for each analyzed variant.

### 3.6. Extraction of Volatile Compounds from Post-Fermentation Product Using the Solvent-Assisted Flavor Evaporation (SAFE) Method

The post-fermentation products obtained by culturing the *G. geotrichum* mold on sour whey and buttermilk media were subjected to extraction to isolate the odor-active compounds. The SAFE method described by Engel et al. [50] was used for this. Samples (50 g) were mixed with dichloromethane (100 mL) and spiked with the internal standard [^2^H_8_] naphthalene (25 μg). The samples prepared in this way were shaken for 2 h on a horizontal shaker to extract volatiles, which were then isolated using the SAFE method. The next step was to dry the obtained extracts under anhydrous sodium sulfate and finally concentrate them to approximately 500 μL using a Kuderna Danish concentrator (Sigma-Aldrich, Poznań, Poland).

### 3.7. GC-O and GC-MS Analysis

#### 3.7.1. Gas Chromatography-Olfactometry (GC-O)

Extracts obtained using the SAFE method underwent GC−O analysis to identify odor-active compounds, using an HP 5890 chromatograph (Hewlett-Packard, Wilmington, DE, U.S.A.) with two columns of different polarities: SPB-5 (30 m × 0.53 mm × 1.5 μm) and SUPELCOWAX 10 (30 m × 0.53 mm × 1 μm) (Supelco, Bellefonte, PA, U.S.A.). In order to select compounds with perceptible odor notes, three panelists sniffed GC-effluent, thus indicating aroma-active regions in the analyzed samples. The effluent was divided between the olfactometry port with humidified air as a makeup gas and a flame ionization port using a Y splitter in GC. The operating conditions for the SPB-5 were as follows: an initial oven temperature of 40 °C (1 min) raised at 6 °C/min to 180 °C and at 20 °C/min to 280 °C. For the SUPELCOWAX 10 column, the operating conditions were as follows: an initial oven temperature of 40 °C (2 min) raised to 240 °C at a 6 °C/min rate and held for 2 min isothermally. SAFE extracts (2 µL) were injected into the GC column using the splitless mode. For all peaks and aroma notes with specific retention times, retention indices were calculated to compare results with those obtained by GC−MS and literature data. For each compound, the retention indices (RIs) were calculated using a homologous series of C7−C24 n-alkanes.

#### 3.7.2. Gas Chromatography-Mass Spectrometry (GC-MS)

After GC-O analysis, odor-active compounds were identified and quantified using a 7890A GC (Agilent Technologies, Santa Clara, CA, USA.) coupled to a 5975C MSD (Agilent Technologies, Santa Clara, CA, USA.). The apparatus was equipped with two columns: SUPELCOWAX 10 (30 m × 0.25 mm × 0.25 μm) and an SLB-5ms (30 m × 0.25 mm × 0.5 μm) (Supelco, Bellefonte, PA, USA). The following operating conditions were used: helium flow of 32.2 cm/s and temperature programs the same as for the GC-O. Mass spectra were recorded in an electron impact mode (70 eV) in a scan range of m/z 33−350. The analyzed volatiles were identified by comparing their mass spectra, RIs, and odor notes on two columns of different polarities with respective standard compounds, National Institute of Standards and Technology (NIST) 09 Mass Spectral Library and literature data.

### 3.8. Quantitation by Stable Isotope Dilution Assays (SIDA)

The SIDA method was used during the quantitation of all identified aroma compounds. Dilutions of stock internal standards of the labeled isotopes for each identified compound (prepared in diethyl ether) were added to samples at similar concentrations to the relevant analyte present in the post-fermentation products. Only in the case of 3-methylbutanal was its direct isotopologue not available; therefore, 2-methylbutanal was used, which in our opinion, is the compound showing the greatest similarity. Odor-active compounds were then extracted and isolated from the samples by the SAFE method, and the obtained extracts were analyzed by GC-MS. During the analysis, the intensity of the individual ions was monitored and is shown in Table 2 for each of the identified aroma compounds. Response factors were calculated in the standard mixture of labeled and unlabeled compounds at a known concentration of 500 ppb for all analyzed volatiles. The response factors were then used to apply corrections. Using the analyte peak areas and the corresponding internal standard, the concentrations for all analyzed aroma compounds were calculated, from which the concentrations obtained for the blank samples were then subtracted.

Finally, the odor activity values (OAVs) were calculated by dividing the concentration of a given analyte by the value of its odor threshold (OT) determined in water.

## 4. Conclusions

In conclusion, determining the optimal conditions for the culture (galactose as the type of sugar in the medium, medium PH at 5.0, and an incubation temperature of 30 °C) allowed for obtaining a high-intensity honey-rose aroma mixture with a pleasant aroma and high desirability, assessed by sensory panelists. While determining the optimal culture conditions, an analysis of the biomass concentration of variants containing different types of sugar in the medium was carried out. The results of this experiment showed that there is no correlation between the concentration of *G. geotrichum* biomass and the intensity of the aroma produced, which allows us to conclude that the sensory profile of the aroma produced is dependent on the culture conditions.

Using GC-O and calculating OAVs, 13 key odorants were identified in products obtained after fermentation of sour whey and buttermilk media by *G. geotrichum* mold. The sour whey post-fermentation product was also characterized by intense fruit-like and sour-like aroma notes. The compounds with the highest OAVs in this variant were the following compounds: 2-phenylethanol (rosy), phenylacetaldehyde (honey), 3-methyl-1-butanol (fruity) and 3-methylbutanal (malty), and isovaleric acid (cheesy). In the variant with buttermilk, the presence of intense caramel-like and buttery notes was found. In this product, the compounds with the highest OAVs were as follows: phenylacetaldehyde (honey), 3-methylbutanal (malty), and 2,3-butanedione (buttery). The presence of the honey-rose aroma of both culture variants is determined by the phenylacetaldehyde and 2-phenylethanol content in the sour whey product and the phenylacetaldehyde in the case of buttermilk.

The obtained results show that the fermentation process of the substrates with sour whey and buttermilk by *G. geotrichum* under certain conditions allows to obtain an aroma mixture with an intense, pleasant aroma of natural origin. This indicates the possibility of using *G. geotrichum* mold to enrich the aroma of foods in many branches of the fermentation industry.

## Figures and Tables

**Table 1:** Key odorants identified in post-fermentation products from sour whey and buttermilk.

Compound ^a^	Odor ^b^	RI-DB 5 ^c^	RI-Wax ^c^	Concentration (µg/kg) ^d^		OT in Water (µg/kg) ^e^	OAV ^f^	
				Sour Whey	Buttermilk		Sour Whey	Buttermilk
2,3-butanedione	buttery	670	991	370	680	15	25	45
acetic acid	vinegar	691	1445	127250	189780	99000	1.3	2.0
3-methylbutanal	malty	695	948	38	36	0.5	76	72
3-methyl-1-butanol	fruity	719	1204	146223	2730	980	149	2.8
butanoic acid	cheesy	836	1620	32040	13030	1000	32	13
isovaleric acid	cheesy	870	1665	19153	6286	490	39	13
3-(methylthio)-propanal	boiled potato	908	1458	13	11	0.43	30	26
dimethyl trisulfide	cabbage	985	1377	0.6	1.5	0.099	6	15
phenylacetaldehyde	honey	1080	1644	1276	3650	4	319	912
furaneol (4-hydroxy-2,5-dimethyl-3(2*H*)-furanone)	caramel	1090	2025	<LOD	<LOD	87		
2-phenylethanol	rosy	1124	1920	125650	4653	240	524	19
ethyl furaneol (2-ethyl-4-hydroxy-5-methylfurane-3(2H)-one)	burnt sugar	1135	2080	28	326	17	1.6	19
(*E*)-2-nonenal	fatty	1160	1527	3.4	3.5	0.19	18	18

^a^ Compounds identified by comparison with reference compounds based on the following criteria: retention index (RI), mass spectra obtained by MS (EI), and odor quality at the sniffing port. ^b^ Odor perceived at the sniffing port. ^c^ Retention indices on DB-5 and SUPELCOWAX 10 columns. ^d^ Mean values based on three replicates with RSD value ≤11%. ^e^ OT: odor thresholds in water [33]. ^f^ OAV: odor activity values calculated by dividing the concentration of an analyte by its odor threshold value.

**Table 2:** Labeled standards and quantitation ions used for SIDA (stable isotope dilution assay) concentration calculations of 13 key odorants present in the post-fermentation products.

Compound ^a^	Quant. Ions ^b^ (*m/z*) ^b^	Labeled Standards ^c^	Ion IS (*m/z*) ^d^
2,3-butanedione	86	[^13^C_4_] 2,3-butanedione	90
acetic acid	60	[^2^H_3_] acetic acid	63
3-methylbutanal	86	[^2^H_3_] 2-methylbutanal	89
3-methyl-1-butanol	70	[^2^H_2_] 3-methyl-1-butanol	72
butanoic acid	73	[^2^H_7_] butanoic acid	77
3-methyl butanoic acid	87	[^2^H_2_] 2-methyl butanoic acid	89
3-(methylthio)-propanal	104	[^2^H_5_] 3-(methylthio)-propanal	107
dimethyl trisulfide	126	[^2^H_6_] dimethyl trisulfide	132
phenylacetaldehyde	120	[^2^H_5_] phenylacetaldehyde	125
furaneol (4-hydroxy-2,5-dimethyl-3(2*H*)-furanone)	128	[^13^C_2_] 4-hydroxy-2,5-dimethyl-3(2*H*)-furanone	130
2-phenylethanol	122	[^2^H_5_] 2-phenylethanol	127
ethylfuraneol (2-ethyl-4-hydroxy-5-methylfurane-3(2H)-one)	125	[^13^C_2_] 4-hydroxy-2,5-dimethyl-3(2*H*)-furanone	130
(E)-2-nonenal	140	[^2^H_2_] (E)-2-nonenal	142

^a^ Quantified compounds; ^b^ Ions used for quantitation of analytes; ^c^ orresponding labeled standards used for quantitation; ^d^ Ions of internal standards (labeled isotopes) used for quantitation.

## Data Availability

The data presented in this study are available on request from the corresponding author.
